# The Effects of Soluble Dietary Fibers on Glycemic Response: An Overview and Futures Perspectives

**DOI:** 10.3390/foods11233934

**Published:** 2022-12-06

**Authors:** Eliana Bistriche Giuntini, Fabiana Andrea Hoffmann Sardá, Elizabete Wenzel de Menezes

**Affiliations:** 1 Food Research Center (FoRC/CEPID/FAPESP), University of São Paulo (USP) Rua do Lago, 250 Cidade Universitária CEP, São Paulo 05508-080, Brazil; 2 Faculty of Science & Engineering, University of Limerick (UL), V94XD21 Limerick, Ireland; 3 Health Research Institute (UL), V94T9PX Limerick, Ireland; 4 Bernal Institute (UL), V94T9PX Limerick, Ireland

**Keywords:** glycemia postprandial, insulin response, glycemic control, viscous fibers

## Abstract

The properties of each food, composition, and structure affect the digestion and absorption of nutrients. Dietary fiber (DF), especially viscous DF, can contribute to a reduction in the glycemic response resulting from the consumption of carbohydrate-rich foods. Target and control of postprandial glycemic values are critical for diabetes prevention and management. Some mechanisms have been described for soluble DF action, from the increase in chyme viscosity to the production of short-chain fatty acids resulting from fermentation, which stimulates gastrointestinal motility and the release of GLP-1 and PYY hormones. The postprandial glycemic response due to inulin and resistant starch ingestion is well established. However, other soluble dietary fibers (SDF) can also contribute to glycemic control, such as gums, β-glucan, psyllium, arabinoxylan, soluble corn fiber, resistant maltodextrin, glucomannan, and edible fungi, which can be added alone or together in different products, such as bread, beverages, soups, biscuits, and others. However, there are technological challenges to be overcome, despite the benefits provided by the SDF, as it is necessary to consider the palatability and maintenance of their proprieties during production processes. Studies that evaluate the effect of full meals with enriched SDF on postprandial glycemic responses should be encouraged, as this would contribute to the recommendation of viable dietary options and sustainable health goals.

## 1. Introduction

According to the Codex Alimentarius Commission (CAC), ‘‘Dietary fiber means carbohydrate polymers with ten or more monomeric units, which are not hydrolyzed by the endogenous enzymes in the small intestine of humans”. This fraction includes carbohydrates polymers that occur naturally in food or are obtained from food raw materials or even synthetic polymers as long as the beneficial health effects have been proven. This definition, published in 2009, which remains in the official CAC labeling guide update [1], considers the physiological effects as well as the chemical nature due to the interdependence between definition and analytical methods that quantify all components of the DF [2]. Although non-plant sources, such as chitin and chitosan, are present in fungi, insects, and invertebrates [3], the most significant sources of DF are of plant origin. Different sources of DF have highly variable compositions and cell wall structures, which include the properties of polysaccharides cell wall matrix such as porosity, cell separation or rupture, and viscosity. These properties affect nutrient bioaccessibility, gastric emptying rate, gastrointestinal transit rate, and the extent of macronutrient digestion and absorption. Fiber plays a role in nutrient encapsulation, where cell walls may remain intact even after mastication and other phases of the digestion process [4]. This is an identified mechanism by which structurally intact plant tissues tend to be digested at a slower rate and, to a lesser extent, attenuates the postprandial increase in glycemia [5]. Some fiber-rich foods, such as those shown in Table 1, can influence gastric function, which includes gastrointestinal transit time and chyme viscosity, thus affecting the flow and behavior of the mixture. The ratio of liquids to solids in a meal influences the time it takes to digest; larger, denser, and/or harder food particles delay gastric emptying, while liquids and small particles move faster through the stomach (during digestive motility). In contrast, larger and heavier particles are collected in the pyloric antrum, with free lipids forming a floating layer on the surface of the bolus [6].

Diabetes care and management evidence analysis provide updated data on the importance of postprandial glycemic targets to delay the onset of clinical complications. The use of modern continuous glucose monitoring devices contributes more robust evidence to this field [7]. Results from a meta-analysis that included 11 randomized clinical trials, with volunteers mainly healthy, mean age ≥19 years, comparing low-GI (≤55) breakfast intervention (s) with high-GI (≥70) breakfast control (s) and evaluated postprandial blood glucose and/or insulin concentrations at 60, 90, and 120 min, showed that low-GI breakfasts significantly reduced postprandial blood glucose concentrations at all time points. This calls attention to the benefits of lowering the breakfast meal GI to provide clinically relevant reductions in acute glucose response [8].

Some factors affect the glycemic response to carbohydrate-rich food, which is the case for soluble dietary fiber (SDF), particularly viscous fibers (that promote the reduction of the gastric emptying rate and/or glucose absorption through the intestinal mucosa) and organic acids and fatty acids (that make the structure more compact by reinforcing the interactions between starch and protein, and by reducing the rate of gastric emptying). Another factor is limiting the accessibility of α-amylase to the starch molecule, which can be obtained during food production by using cereal varieties with high amylose content, whose linear structure has a lower hydrolysis rate than the branched form of amylopectin. Another option is incorporating cereal grains into products because these food particles in the gastrointestinal tract are preserved and emptied more slowly from the stomach, reducing the glycemic response [9].

**Table 1:** Examples of soluble dietary fiber (SDF), characteristics, and sources.

SDF	Main Polymer	Main Monomers	Linkage	Viscosity	Source	Reference
	Alginate	Mannuronic acid and guluronic acid	β-(1,4) D-mannuronic acid or α-(1,4) L-guluronic acid	Viscous	Cell walls from brown seaweed (*Phaeophyceae*)	Garcia-Cruz et al., 2008 [10]
	Arabinoxylan	Arabinose and xylose	β-D-(1,4) xylose	Viscous	Cereal grains	Elleuch et al., 2011 [11]
Psyllium	Arabinoxylan	Arabinose and xylose	β-(1,4) linked D-xylopyranosyl residues (Polymer of arabinoxylans with 1,4 and 1,3 linkages)	Viscous	*Plantago ovata* (seeds)	Fischer et al., 2004 [12]
	β-glucan	Glucose	β-(1,4) and β-(1,3) glucose	Viscous	Oat, barley, yeast, algae	Elleuch et al., 2011 [11]
	Galactomannan	Mannose and galactose	β-(1,4) mannopyranose, β-(1,6) D-galactopyranosyl	Viscous	Seed gums from leguminous plants and microbial sources (yeast and fungi)	Srivastava; Kapoor, 2005 [13]
Guar gum	Galactomannan	Mannose and galactose	β-(1,4) linked D-mannopyranosyl units with α-(1,6)-linked D-galactopyranosyl residues as side chains	Viscous	*Cyamopsis tetragonolobus* shrub	Mudgill et al., 2018 [14]
	Inulin	Fructose, glucose	β-(2,1) D fructosyl-fructose	Non-viscous	Chicory (root), onion, garlic	Elleuch et al., 2011 [11]
Konjak gum	Glucomannan	Glucose e mannose	β-(1,4) D-glucose and D-mannose, β-(1,6)-glucosyl	Viscous	*Amorphophallus konja* (tubérculo)	Chua et al., 2010 [15]
	Pectin	Galacturonic acid and rhamnose,	α-(1,4) linked D-galacturonic acid, (1,2) linked L-rhamnose	Viscous	Citrus peel, apple pomace, sugar beet pulp	Voragen et al., 2009 [16]
	Pullulan	Glucose	three α-1,4-linked glucose polymerized by α-1,6 linkages on the terminal glucose, resulting in a stair-step structure	Low viscosity	Secreted by the fungus *Aureobasidium pullulans*	Wolf et al., 2003 [17]
	Resistant maltodextrin (RD)	Dextrose	α-(1,4) D-glucose, α-(1,6) D-glucose	Low viscosity	Heat and enzymatic treatment of starch	Elleuch et al., 2011 [11]
Soluble corn fiber	RD	Dextrose, fructose	α-(1,4) glucose α-(1,6) D-glucose	Low viscosity	Product from enzymatic hydrolysis of cornstarch	Harrison; Hoffman, 2007 [18]
Nutriose	RD	Dextrose	α-(1,4) glucose α-(1,6) D-glucose and α-1,6 and (or) β-1,6; a-1,2 and (or) β-1,2; α-1,3 and (or) β-1,3; and β-1,4	Non-viscous	RD obtained from wheat, maize, or other edible starch	Hobden et al., 2021 [19] Li et al., 2010 [20]
Polydextrose		Glucose	α- and β-(1,2) (1,3) (1,4) (1,6) D-glucose, with a predominance of α and β (1.6)	Viscous	PDX is a highly branched, randomly bonded synthetic glucose polymer	do Carmo et al., 2016 [21]
Xanthan gum		Glucose, mannose, glucuronic acid	β-(1–4) D-glucose	Viscous	Fermentation by *Xanthomonas campestris*	Bhat et al., 2022 [22]

In recent decades, several studies have demonstrated that extracts rich in polyphenols are also effective α-amylase inhibitors that reduce postprandial blood glucose [23,24,25]. Produced by the pancreas and salivary glands, α-amylase catalyzes the hydrolysis of α-(1,4)-D-glucan linkages from starch and other glucose polymers. Many flavonoids have already been tested for the inhibitory activities of α-amylase, and according to Mahmood (2014) [23], luteolin, luteolin 7-O-glycoside, and daidzein appear to be the most efficient inhibitors. Consumption of native Brazilian fruit juices (300 mL), which are rich in flavonoids, reduced the glycemic response of bread (50 g) from 11% to 64% after consumption by 23 healthy volunteers in a short-term trial [24]. Polyphenols are compounds associated with DF, and the association of these two components could contribute to reducing the glycemic response of foods. However, the processing and structural alteration of food sources of DF is a critical factor that can alter intestinal digestion and the bioavailability of intracellular nutrients, which can undergo oxidation, for example [25].

A systematic review study with a meta-analysis of RCT analyzed the effect of dietary fiber (DF) intake on glycemic control in patients with type 2 diabetes (T2DM), using glycated hemoglobin (HbA1c) and fasting plasma glucose as parameters, evaluated at the beginning and end of the study. Data from 11 studies with a duration range of 8−24 weeks, n = 605 patients, were used. Diets rich in DF, which include fiber-rich foods (up to 42.5 g/day; four studies) or supplements containing SDF (up to 15.0 g/day; nine studies) reduced HbA1c values by ~5%, which was considered clinically relevant as it is similar to the proportion obtained by some medications for T2DM. The 9.97 mg/dL reduction in fasting plasma glucose led the authors to consider SDF as an adjuvant tool in the treatment of patients with T2DM [26].

Adding DF to solid and liquid foods rich in carbohydrates can promote significant reductions in postprandial glucose absorption. However, palatability issues at the concentrations needed to see the beneficial effect may limit its applications as a functional food ingredient. At the same time, food processing can cause changes in the physicochemical properties of DF and negatively impact the viscosity of soluble fractions, reducing their effectiveness [27].

Oat β-glucan was extruded under different conditions, thus resulting in a range of molecular weight fractions and distinct viscosities. Microscopic examination showed that more severe extrusion conditions cause depolymerization, where the integrity of the cell walls was lost, and β-glucan dispersed throughout the cereal. Differences in the hardness and density of the extruded cereals were also evident as the molecular weight decreased [28].

An interesting study using several imaging techniques (X-ray diffraction, micro-X-ray microtomography, and electronic microscopy) evaluated four food products produced with different processing technologies (extruded products, rusks, soft-baked cakes, and rotary-molded biscuits). It was possible to observe that the rotary-molded process preserved the higher content of slowly digestible starch and its crystalline structure, thus resulting in a lower glycemic and insulinemic response [29].

### Mechanisms for Reducing the Glycemic Response

According to McRorie Jr. and McKeown (2017) [30], the efficacy of soluble dietary fiber (SDF) in glucose and insulin metabolism seems to be proportional to the viscosity of hydrated fiber, which has been observed in short-term studies [31]. Several clinical studies lasting a few months found that consumption of a viscous, soluble fiber supplement (for example, gel-forming fibers such as psyllium and guar gum) given with meals can improve glycemic control [12,30,32,33,34]. This involves reducing fasting plasma levels of glucose, insulin, and HbA1c, which are observed in individuals at risk of developing T2DM and patients being treated for T2DM. A mechanism to improve glycemic control with an SDF supplement is to significantly increase the viscosity of the chyme in a dose-dependent manner [35]. Viscosity can delay gastric emptying, and the absorption of glucose in the small intestine slows down; in addition, there are increases in the viscosity of the unstirred layer [31]. The effect of viscosity on glucose release was studied in a well-controlled in vitro model (TIM-1-system). Three different conditions (∼1 mPa·s, ∼15 mPa·s, and ∼100 mPa·s) were tested, and when the bolus viscosity increased from ∼1 mPa·s to ∼15 mPa·s, the maltodextrin to glucose conversion was reduced by 35%, but no effect was present in the remaining increment [36].

The increasing viscosity slows the interaction between digestive enzymes and nutrients and, consequently, the breakdown of nutrients into components that will be absorbed in the brush border, including glucose [37]. A more viscous chyme, due to the presence of SDF, slows digestion and absorption; hence, nutrients that would usually be absorbed at the beginning of the small intestine can reach the distal ileum, where they are usually minimally present [38]. Nutrients administered to the distal ileum can stimulate mucosal L cells to release glucagon-like peptide (GLP-1) into the bloodstream. GLP-1 has a short half-life (~2 min) associated with reduced appetite, stimulating the growth of pancreatic beta cells (insulin-producing cells), improving insulin production and sensitivity, and decreasing glucagon secretion (the peptide that stimulates glucose production in the liver) [37]. The presence of macronutrients in the distal ileum can also stimulate the phenomenon of the ileal brake, a mechanism mediated by the ileal hormone peptide YY and GLP-1, in a combination of effects that influence the digestive process and food consumption [38]. The ileal brake phenomenon can effectively delay gastric emptying and small bowel transit, attenuating nutrient loss to the large intestine [37,38]. It is important to note that viscous fiber can only delay but not reduce total nutrient absorption [39], which can occur throughout the entire small intestine.

The mechanism related to the viscosity of SDF is well-documented [6,31,36,39]. Still, the presence of fermentable soluble fibers may also be responsible for the reduction in blood glucose and postprandial insulin levels [40], and the decrease in blood glucose and insulin peaks after the first and second meals [41]. This effect may be due to the reduced digestibility of soluble fiber, such as soluble corn fiber [40], and short-chain fatty acids (SCFAs) production during colonic fermentation. SCFAs act on intestinal endocrine cells and/or in neurons of the enteric nervous system to alter gastrointestinal motility and secretion [42].

SCFAs also act as signaling molecules, activating G protein-coupled receptors (GPCRs), especially GPR41 and GPR43 on the brush border membrane. They stimulate the release of GLP-1 and peptide YY by enteroendocrine cells [43], which are hormones also stimulated by macronutrients in the ileum, with the effects described above. The Na^+^ glucose cotransporter SGLT1 facilitates intestinal absorption of glucose and galactose. In contrast, fructose uptake is facilitated by GLUT5, transporters expressed on the apical surface of enterocytes from the small intestine [44]. If an SDF delays the arrival of sugars in the intestinal lumen, it limits their accessibility to their respective enterocyte receptors. In that case, the absorption of these sugars and the postprandial glucose response will be delayed. In addition, SGLT1 stimulation can be reduced by releasing GLP-1 and peptide YY due to the presence of glucose in the most distal region of the intestine, with a higher prevalence of L cells.

The present review focuses mainly on demonstrating with clinical trials the potential of less studied types of soluble dietary fiber in reducing the glycemic response.

## 2. Methodology

Searches were carried out for scientific articles from the 1970s onwards with several databases, such as PubMed, Science Direct, and Google Scholar. The keywords utilized were: “soluble dietary fiber (and fibre)”, “viscous fiber (and fibre)”, “glycemic (and glycaemic) response”, “glycemic (and glycaemic) index”, “glycemic (and glycaemic) control”, and different fiber type names: xanthan, beta-glucan, guar gum, alginate, arabinoxylan, pullulan, fructan, resistant dextrin, and others.

Studies with inulin were not included since the glycemic response potential of this fiber has already been consolidated. Data from GI prediction studies (in vitro) and animal trials were not included.

There were no exclusions from clinical studies with SDF as long as they were performed with only soluble dietary fiber alone or with a dietary source of soluble fiber. Studies with healthy volunteers or with some morbidity (diabetes, overweight), regardless of number, sex, and age, and also of the type of outcome (glycemic response, insulin response, area under the curve, blood glucose and/or insulin resistance, fasting blood glucose, HbA1c, GI determination, peak blood glucose or insulin, etc.) were included. Review studies and meta-analyses were included in the introduction for discussion or summarizing a concept.

## 3. Human Studies with Dietary Fibers and Glycemic Response

Several studies, whose descriptions are summarized in Table S1 (Supplementary Material), have been conducted with different types of fibers to confirm the efficient performance of DF on the postprandial glycemic response. Inulin is one of the most studied fibers for glycemic control, although the results are not always concordant. Inulin, a non-viscous SDF, is a fiber fermented in the intestine that delays the rate of gastric emptying, thereby reducing glucose absorption and postprandial blood glucose elevation. Propionic acid, a fermentation product, may reduce hepatic gluconeogenesis and affect hepatic glucose metabolism [45]. A review involving 33 randomized controlled studies (n = 1346 volunteers) with dose–response models that considered the duration of supplementation, verified an association between this type of fiber and blood glucose parameters. Inulin supplementation significantly reduced fasting blood glucose and insulin, HbA1c, and homeostasis model assessment-insulin resistance (HOMA-IR). These effects were even more pronounced in patients with prediabetes and type 2 diabetes; for this population, the authors recommend daily doses of 10 g and for periods of at least six weeks [45].

The most relevant data from clinical studies related to the intake of different SDFs and their impact on the glycemic response will be presented below. The characteristics and sources of these SDF are presented in Table 1.

### 3.1. Alginate

According to El Khoury et al. (2014), the consumption of alginate in drinks diluted in water and sugar reduces the glycemic response and the sensation of appetite. This study investigated the effect of alginate when added to chocolate milk, which has been widely criticized for increasing plasma glucose more than flavorless milk. CM with 2.5% alginate reduced glucose peak by approximately 32% at 30 min compared to CM with 1.25% alginate and by 46% when compared to alginate-free CM. The three initial alginate treatments reduced postprandial glucose and total glucose responses compared to CM alone. The insulin peak at 30 min was decreased by 46% after CM with 2.5% alginate compared to alginate-free CM. Preload consumption of CM with added sodium alginate decreased glycemia, insulinemia, and appetite after a meal [46].

Volunteer males took a preload drink containing either ionic-gelling alginate (1.5 g of sodium alginate) or an acidic-gelling control before eating lunch. The mean postprandial peak blood glucose rise (PBGR) and glycemic area under the curve (AUC) were 14% and 52% lower, respectively, after consumption of the ionic-gelling alginate drink compared to the control drink. Body composition affected the postprandial glycemic response but did not interfere with the effects of alginate [47].

The study by Jensen et al., (2012) [48] evaluated the effect of ingestion of preload beverages with added alginate with different volumes, ingested 30 min before receiving the fixed breakfast and again before an ad libitum lunch, on the postprandial glycemic response of healthy young volunteers. The preload of high volume (HV)-alginate (500 mL, 15 g) provided a 40% decrease in total AUC glucose response compared to HV-control. This effect was not observed in the low volume (LV)-alginate (330 mL, 9.9 g) and LV-control. Another study conducted with T2DM men, which included meals consumed with 5 g of sodium alginate (algae isolate, 75% soluble fiber), observed a reduction in the AUC of plasma glucose, insulin, and C-peptide by 31%, 42%, and 35%, respectively. A negative correlation was observed between the increased gastric emptying half-time (min) and the decreased postprandial glycemic response induced by the fiber added to the meal [49].

The peak blood glucose levels, when compared to the control meal without alginate, was reduced by 11% and 15%, respectively in the groups that received noodles containing 5% or 8% calcium alginate; in addition, there was a 15% and 21% reduction in AUC. It is essential to note that a questionnaire indicated that the addition of alginate did not affect the acceptability of the noodles [50]. The results of a study with healthy adults showed that the consumption of sugar beverages with only the addition of soy protein isolate (SPI) at pH 6 and 7, and two others mixed SPI with alginate significantly reduced peak plasma glucose concentration at 30 min (33.4%, 36.3%, 53.2%, and 58.5%, respectively) in relation to control, and showed a significant reduction in the peak glucose between SPI with and SPI without alginate. There was also a significant reduction in glucose AUC and peak insulin concentration compared to beverage control. The authors considered that interactions between protein and alginate during digestion, forming a gel, may positively affect the postprandial glucose response [51].

### 3.2. Arabinoxylan and Psyllium (Arabinoxylan Source)

Arabinoxylan (AX), hemicellulose present in the cell wall, is the main fiber component in cereal grains. For a study by Lu et al., (2000) [52], arabinoxylan was extracted from wheat bran to produce a fast-fermented arabinoxylan-rich soluble fiber, which was used in bread (0, 6, and 12 g of AX). A significant reduction in blood glucose and peak plasma glucose were observed. The AUC was reduced by 41.4% and 20.2% in relation to glucose and 32.7% and 17.0% in relation to plasma insulin for doses in a dose–response relationship when consumed in meals (75 g of available carbohydrates (50 g of starch + 25 g of sugar); 10 g of protein; 14 g of lipids) with doses of 0, 6, and 12 g of AX. At the same time, the AX-rich fiber bread was considered palatable and well-accepted by volunteers. The authors attributed these results to delayed glucose absorption due to the viscosity that slowed the gastric emptying rate and the small intestine’s motility [52].

In a 5-week intervention study with T2DM, fasting plasma glucose and insulin and oral glucose tolerance (OGT) among other assessments were determined before and after an intervention with 15 g/day AXN. The volunteers that consumed the AX diet presented a significant reduction in fasting blood glucose and insulin and 2h-OGTT, demonstrating that AX fiber may potentially improve glycemic control [53].

The AX enrichment in breakfast did not significantly alter postprandial plasma glucose levels, but it did decrease insulin levels. However, the insulin responses were not significantly different in individuals with a normal glycemic response. In young and healthy individuals, adding 6 g of AX to the offered meal did not change the glycemic response parameters [54].

Serum glucose and insulin increased rapidly after the consumption (30 min) of liquid meals with and without a supplement of 15 g AX by overweight volunteers, but the postprandial response was lower (AUC_240 min of serum glucose and insulin) [55]. Moreover, fasting serum glucose was lower after 6 weeks of consuming AX compared to the placebo; however, AX did not affect fasting insulin levels [56].

A meta-analysis of psyllium supplementation included 35 studies with randomized, controlled, short- or long-term trials in a healthy population with prediabetes or T2DM (n = 6 to 225 participants). Psyllium intake had no impact on euglycemic populations, and improvement was modest in pre-diabetics; however, psyllium consumption reduced fasting and postprandial glucose, as well as HbA1c in individuals treated for T2DM [57].

After 8 weeks of intervention with 10.5 g psyllium supplementation, T2DM volunteers in the intervention group showed a significant reduction in fasting blood glucose and insulin levels, HbA1c, HOMA-IR, and HOMA-β% compared to the control group that continued with a regular diet without any supplementation. The authors attributed the improvement in insulin sensitivity, delay in the entry of glucose into the bloodstream, and decrease in the postprandial rise of blood glucose to the viscosity of soluble fibers in the gastrointestinal tract [33]. In a study with 10 g psyllium ingestion (pre-mixed in sugar-free cookies) for 12 weeks, twice per day, fasting glucose and HbA1c of TD2M volunteers were reduced in the psyllium group but not observed in the placebo group, which received sugar-free cookies. Fasting plasma glucose decreased from baseline in the psyllium group but not HbA1c [58]. However, in another similar study, the change in fasting plasma glucose from baseline was different between the psyllium, flaxseed, and placebo groups and persisted even after four weeks; the difference observed for HbA1c was not significant [34].

T2DM volunteers ingested two doses of psyllium (6.8 and 13.6 g/day) for 12 weeks. Both doses significantly reduced fasting blood glucose compared to the placebo at weeks 4, 8, and 12. Psyllium, at a dosage of 13.6 g, significantly reduced HbA1c at eight weeks, and both doses significantly reduced HbA1c at 12 weeks compared to the placebo. The authors consider psyllium an effective co-therapy to improve glycemic control in T2DM patients [35].

The addition of 17.14% psyllium promoted 25% GI and 39% GL reductions, resulting in a low-GI and low-GL food with suitable acceptability, compared to gluten-free bread (GFB; 2.51 g DF/100 g food) with medium GI and GL values [59]. With the replacement of rice flour for chickpea flour (CF), it was possible to reduce GI by 21% and GL by 49% and obtain a GFB with low GI and GL, while the combined effect of CF and psyllium (5, 5%) reduced GI by 25% and GL by 52%, similar to GFB enriched with 17.1% psyllium. These replacements also caused a reduction in the glycemic response curve in 30, 45, and 60 min intervals compared to bread made with rice flour. In addition, replacing rice flour with CF and psyllium increased the satiety index [60].

### 3.3. Arabinoxylan (AX) and β-glucan

Rye kernels (RK) are rich in AX and DF, which can be found in soluble and insoluble forms [61]. Different types of DF in bread (with a portion of 50 g of available carbohydrates) were consumed by individuals with metabolic syndrome at varying doses, AX (AX = 7.1 g); β-glucan (BG) (AX = 2.6 g); RK (AX = 6.1 g); and white bread (WB) (AX = 1.1 g). Volunteers that consumed BG and RK presented reduced glucose iAUC at 120 min compared to the WB group. AX reduced the glucose peak value compared to WB. When comparing insulin responses at 120 min, the lowest value found was for RK, and BG achieved a more significant reduction than AX, similar to the control [62]. A similar study was carried out with semolina porridge with added AX concentrate (AX; AX = 3.5 g), rye grains (RK; AX = 4.7 g), or AX concentrate combined with rye grains (AXRK; AX = 4.4 g); semolina porridge was used as a control (SE; AX = 0.9 g). The AXRK meal reduced the glucose iAUC response, peak glucose, and insulin iAUC compared to the SE control meal. AX alone did not produce a significant response in relation to glucose or insulin iAUC, but produced a relative reduction in the glucose iAUC in relation to SE [63]. Thus, AX can contribute to the glycemic response in an isolated and concentrated way, but these two studies found better results when AX was added in the form of rye kernels.

Short and medium-term studies of the blood glucose and insulin response to AX were conducted, and it is hoped that additional long-term studies will be conducted to investigate the regular intake of this type of dietary fiber to understand its beneficial effects on the human body.

### 3.4. β-glucan

β-glucan, a soluble fiber in oats and barley, has been demonstrated to attenuate postprandial glycemic and insulin responses. β-glucans are non-starch polysaccharides composed of glucose molecules in linear polymers with β(1,4) and β(1,3) bonds at an approximate distribution of 70% to 30%, respectively. A common barley cultivar has 68% starch and 5% β-glucan, but the cultivar Prowashonupana (PW) is barley with low starch content (31%) and high β-glucan content (18%).

In the study by Liljeberg et al., (1996) [64], the average amount of β-glucan (dry basis) in raw oats was 4.0 g/100 g, in common barley 4.7 g/100 g, and in PW barley 17.6 g/100 g. They have been added to porridges and bread for glycemic index (GI) and insulin index (II) determination. The values of GI and II were GI = 106 and II = 100 for oatmeal porridge; GI = 97 and II = 103 for common barley porridge; GI = 77 and II = 73 for porridge with 50% PW; GI = 71 and II = 46 for bread with 50% PW, GI = 61 and II = 43 for bread with 80% PW. The glycemic response was significantly lower at 30 and 45 min for PW foods. Insulin was also lower for these foods at 30 min. Although all foods are suitable sources of DF, only foods produced with barley of the PW genotype could alter the glycemic response of volunteers [64].

Bread with wheat flour (reference food) and others with added 50% common barley and 35%, 50%, and 75% of PW barley were studied too. The GI (83%) and II (81%) of bread with added common barley showed no significant difference compared to typical bread (GI = 100 and II = 100), while bread with 35%, 50%, and 75% of PW presented respectively GI values equal to 75%, 65%, and 55%, and II of 74%, 65%, and 68%, which were significant for the concentrations of 50% and 75% PW. The authors consider this due to the viscosity of the PW fiber rich in the β-glucan present in these breads, which can reduce the motility of the luminal content and/or thicken the stationary water layer, with a consequent reduction in the capacity to absorb carbohydrate [65].

No differences were observed in GI and II between muesli (raw rolled oats) and oat porridge (boiled rolled oats) in relation to white bread when they were consumed by healthy men; however, kernel porridges produced low glucose and insulin responses, with GI = 67 for wheat kernel porridge and GI = 60 for oat kernel porridge, both with II = 68%. Neither incomplete gelatinization in rolled oats nor the natural viscous dietary fiber in oats, β-glucan, was able to affect postprandial glycemia, but the presence of intact grains in meals significantly decreased metabolic responses [66].

According to Cavallero et al., (2002) [67], data confirm the dose–response hypothesis of β-glucan concerning glycemic response. A control bread and three β-glucan-enriched breads were produced: the β-glucan content was 0.1 g/100 g for wheat flour bread (100%), 2.4 g/100 g for 50% whole-grain barley flour (BF) bread, 4.3 g/100 g for bread with 50% sieved fractions (SF), and 6.7 g/100 g for bread with 20% water extraction fraction (WF). The WF process had increased the β-glucan content (33.2%) compared to the barley sieved fraction (8.5%). Bread with BF had GI = 99 and bread with SF had GI = 82, with no significant difference in relation to GI = 100 for wheat flour bread (100%). The incorporation of 20% flour with high (1→3,1→4)-β-glucan barley fraction, by water extraction (WF), reduced GI to 72 and achieved the best scores in other attributes [67].

Oat granola underwent a β-glucanase treatment to produce products with low, medium, and high molecular weight, which presented different viscosity, solubility, and rapidly digestible starch percentages. PBGR and iAUC were lower in the formulation with 40 g of total available carbohydrates (TAC) than in the formulation with 60 g of TAC. The formulations with 60 g TAC with high molecular weight (MW) had lower peaks and iAUC in relation to the wheat or oat-based granola (0.6 and 6.2 g of β-glucan) and control. The formulations with 40 g TAC with high MW had a lower peak in relation to the other 40 g MW and control. The iAUC high MW and medium MW were lower in relation to the 40 g with low MW and control. β-glucan with low MW presented results similar to the control (wheat-based product). When the β-glucan/starch ratio was 1.6:10 instead of 1.1:10, the reduction in PBGR and iAUC was significantly greater. As the viscosity of β-glucan extract increased in vitro, starch digestibility in vitro was reduced and the glycemic response was lower [68].

The viscosity of oat β-glucan can be altered by concentration in liquid foods. β-glucan drinks with the same molecular weight but different volumes had different viscosity (>9-fold difference) but did not change the glycemic response; there was no difference in AUC of glucose between drinks. However, the effects of β-glucan on peak glycemic response were altered by changes in molecular weight; the more viscous high MW drink achieved greater reductions in peak glycemic response than the low MW drink. Changing viscosity through volume did not alter the effect of β-glucan on glycemic response, but volume did affect the pattern of the glycemic response curve through its effect on gastric emptying. The physical presence of β-glucan and its high MW was more important in decreasing the glycemic response than volume [69]. This study indicates that the initial viscosity of the solution appears to have no relationship with the viscosity resulting from the action of amylase after consumption and that the digestion process must adjust the viscosity of the chyme, which thus, affects gastric emptying and glucose absorption [6]. Volunteers consumed two types of breakfasts, with the difference being the addition of oat bran to the test meal compared to the control. Intake of 4 g of high MW oat β-glucan modulated glycemic response and parameters of satiety and hunger. Blood glucose was reduced at 30 min and plasma insulin at 30 and 60 min, as well as the insulin AUC. The authors observed that the test meal rich in oat β-glucan showed substantially higher viscosity than the control meal, evaluated by shear rate representative of gastric conditions. They concluded that the higher viscosity could delay gastric emptying and subsequent glucose absorption, although they did not directly assess this [70].

There was no difference between the control meal and different oat β-glucan (OBG) (4 g β-glucan) preload treatments for peak glucose increase and iAUC in 0–120 min, but a difference was observed for glucose iAUC in 0–45 min and time to peak glucose for four products with higher molecular weight OBG; the highest iAUC in 0–45 min was associated with the lowest MW and viscosity. In this study, OBG (low MW) consumed as preload seemed to accelerate the rise in blood glucose but reduced the time it took for glucose to peak and it did not affect glycemia peak or iAUC. The authors concluded that the results were not as expected and may be attributed to the small load of 4 g of OBG, possibly because this viscous preload was diluted by oral and gastric secretions while the bread was ingested afterward [71].

For β-glucan and postprandial glycemic response, several studies evaluated oats and barley, which are suitable sources of this SDF; however, barley of the Prowashonupan (PW) genotype (18% β-glucan) provided the most effective reduction in the postprandial response of glucose and insulin compared to the common cultivar (5% β-glucan), even when added to bread. Studies show that the physical presence of β-glucan with whole grain and of high molecular weight is essential for decreasing the glycemic response.

The effect of kilning, tempering, microwaving, cooking, soaking, and flaking on oat β-glucan viscosity were studied to test oatmeal products containing low, medium, and high β-glucan viscosity for their effect on postprandial glycemic response in volunteers, but the increase in viscosity of the oat β-glucan by processing did not have any impact on postprandial glucose [72]. Premeal and post-meal blood glucose levels were lowest for breakfast, lunch, and overall, when adolescents with T1DM consumed oat flakes containing 6 g/day of β-glucan for one week compared to oat flakes containing 3 g/day of the β-glucan or a standard diet. Peak blood glucose levels were also lower at breakfast, lunch, dinner, and overall, with 6 g/day of β-glucan. In addition, peak, mean, daytime, and nighttime blood glucose levels were the lowest with the intake of 6 g/day of the β-glucan for 1 week. Oat flakes containing 6 g/day β-glucan also showed delayed peaks for all time points compared to earlier phases of the study with 0 g/day and 3 g/day β-glucan [73].

Preloads of oat bran (4.5, 13.6, or 27.3 g) containing 22% of high MW oat β-glucan (O22), mixed in water before a test meal of white bread, showed a significant effect on blood glucose at 15, 30, 45, and 120 min. Blood glucose with 27.3 g preload oat bran was significantly lower than those after 0 g and 4.5 g at 15 min, and after intake of 27.3 g, the glycemia was significantly lower than after the intake of 13.6 g, which was significantly lower than the 0 and 4.5 g doses at 30 min. Blood glucose concentration at 45 min after intake of 27.3 g was significantly lower than those after the other doses. There was a significant effect of dose on blood glucose AUC when compared to bread AUC. The responses for the 4.5, 13.6, and 27.3 g doses were 95.0%, 90.3%, and 76.3%, respectively, with the latter being the only significant [74]. Compared to breakfast with cream of rice (CR), volunteers who consumed breakfast plus oat 4 β-glucan (4gOBG) presented significantly reduced glucose AUC 2 h and insulin AUC 2 h after a subsequent meal (pizza). Glucose peak rise and mean glucose iAUC 2 h was lower after oat 2 β-glucan (2gOBG), 4gOBG, and 4gloMW (oat bran plus β-glucanase to reduce OBG MW and viscosity compared to 4gOBG) than after CR. The glucose peak was lower after 4gOBG compared to 2gOBG and 4gloMW. Mean glucose iAUC 3 h after 4gOBG was lower than after CR. After consumption of CR, 2gOBG and 4gloMW, insulin liberation was similar, considering iAUC 2 h, iAUC 3 h and peak and all greater than after 4gOBG [75].

The DFs were added individually and in combination to produce samples with large differences in structural/rheological properties in pasta and to see if this could alter the glycemic response. Pasta (15% DF) containing barley balance (25% β-glucan and 13% other DF) and in combination with 7.5% barley balance and 7.5% psyllium seed husk (selected for having overall sensory acceptability) demonstrated a reduction in the glycemic index (GI = 33 and 35%), which was lower than the control food (durum wheat semolina pasta) with GI = 52. Different elastic properties were observed between 25 and 95 °C due to the number of interactions between the components and the swelling of gelatinized starch granules. According to the researchers, the changes in viscoelastic properties were likely due to interactions between fiber and gluten and/or aggregation or association of polysaccharide chains [76].

When different amounts of cereal β-glucan (low: 0.8 g, medium: 3.2 g, and high: 6.6 g) were consumed in the evening meal for three days, only the medium intervention was able to reduce blood glucose and insulin during glucose tolerance (OGT) testing in the following day when compared to baseline. The authors considered the beneficial effects of β-glucan on glycemic regulation the following day are not dependent on viscosity [77]. However, some studies confirmed the dose–response hypothesis of β-glucan in relation to the glycemic response postprandial [67,73].

### 3.5. Pleurotus *spp.*

Edible fungi have been considered sources of DF and β-glucan [78]. *Pleurotus sajor-caju* (PSC) powder has been added to wheat-based foods to provide more DF. PSC is an easy-to-grow, consumable gray oyster mushroom with a pleasant taste and interesting pharmacological properties. Patients who had recently been diagnosed with type 2 diabetes were randomly divided into three groups that received biscuits made with either ajwain (carom seeds), ajwain + mushroom, or mushroom biscuits (*Pleurotus* spp), respectively. Ajwain is a small, oval, seed-like fruit eaten roasted and often sprinkled on biscuits. After 3 months, there was a reduction in fasting glucose and HbA1c in the ajwain + mushroom group and the mushroom group, both in relation to the baseline and in relation to the ajwain group, which had no differences relative to the baseline [79].

*Pleurotus ostreatus* (P.o) and *Pleurotus cystidiosus* (P.c) were lyophilized, and the powder was diluted in an oral solution to be consumed at a dose of 50 mg/kg body weight, followed by a glucose load (75 g in 300 mL of water) by healthy volunteers and patients with T2DM in a controlled diet. P.o and P.c produced a significant reduction in fasting and postprandial serum glucose levels in healthy volunteers and patients with diabetes, with increased serum insulin levels for T2DM patients [80].

Fresh samples of PSC were dried and ground, and different amounts of this powder were added to the biscuits. The original DF content in the biscuit was 3.37% (β-glucan = 0.12%), and the presence of PSC powder in 4% PSC, 8% PSC, and 12% PSC concentrations, increased the DF content to 6.19%, 8.62%, and 9.84% (β-glucan = 1.06%, 1.29%, and 1.78%), respectively, and reduced the paste viscosities and the starch gelatinization enthalpy value of the biscuits. The addition of PSC powder also interfered with the integrity of starch granules, reducing sizes and inducing uneven, spherical shapes, which resulted in the reduced susceptibility of starch to digestive enzymes. The iAUC of all biscuits was reduced in relation to glucose, but the biscuits with the addition of 8% also presented a difference in relation to the biscuits with 0% and 4% PSC. The starch hydrolysis rate significantly reduced the GI of the biscuits; the addition of 4, 8, and 12% PSC resulted in a GI of 52.0, 49.0, and 47.4, respectively, and the biscuits with 4% exhibited a significant difference in relation to the concentrations of 8% and 12%; there was no difference between these two levels of addition. In the sensory evaluation, the biscuits with the most PSC had the lowest scores, possibly due to the higher degree of firmness, stronger aroma, and darker color. The authors conclude that incorporating 8% PSC powder into biscuits (GI = 49) can be an effective way to develop a nutritious biscuit with low GI without compromising its desirable sensory properties [81].

After regular intake of *Pleurotus ostreatus* (3 g/day, in 3 divided doses) for 3 months, fasting plasma glucose from baseline decreased, including the HbA1c%, and had no detrimental effect on the renal system [82]. In an acute study, *Pleurotus eryngii* (β-glucan = 4.5 g) reduced the postprandial glucose only when compared with the control [83]. Tortillas with different levels of *Pleurotus sajor-caju* powder (PSP) (0%, 5%, 10%, and 15%) to partially replace wheat flour were investigated and, in the sensory evaluation, the tortilla with 15% PSP was the most accepted by the sensory panel. The addition of 15% PSP in the tortilla increased the dietary fiber content (13.62 g/100 g), and β glucan (1.21 g/100 g) compared to the control tortilla (TDF = 9.31 g and β glucan = 0.69 g per 100 g) and had a low GI (53) while the control tortilla had an intermediate GI value (58). Both test foods were significantly different for GI, iAUC, and blood glucose levels were significantly lesser at 15, 30, 45, and 60 min. It is possible that β-glucan decreases postprandial glucose response in the gastrointestinal tract due to high viscosity but also reduces starch breakdown by α-amylase [84].

According to the studies presented, *Pleurotus* spp. powder added to food was able to affect parameters related to the glycemic response (fasting glucose, iAUC, GI, Hb1ac) both in healthy volunteers and in diabetic patients, but depending on the amount added, the texture, color and aroma of products may be compromised.

### 3.6. Guar Gum and Galactomannans

Some studies on guar gum, either alone or in a fiber blend, with doses ranging from 5.0 to 15 g, ingested with bread, soup, or different controls, showed a reduction in peak blood glucose rise (PBGR) and/or AUC, and some studies reported similar results for insulin [85,86,87,88,89]. Studies show that these effects of guar gum are influenced by the consistency of the food (better in liquid than solid form), and particle size with palatability is related to the dose consumed. Unfortunately, adding this fiber presents palatability problems at the concentrations necessary to promote physiological effects, which may limit its application in industrialized products [90].

A three-hour double-blind glycemic response study was performed in which participants consumed four types of meals that contained 50 g of available carbohydrate, white bread (25 g) + 25 g of a drink with maltodextrin (reference food), or + drink with maltodextrin and with 5 g of guar gum added or 5 g of fructose added or 5 g of guar gum + 5 g of fructose added. The incremental peak glucose was reduced with the ingestion of the beverage containing added guar gum and guar gum + fructose. The AUC was reduced for guar gum ingestion alone [90]. Formulations that mix a source of starch and certain types of fiber can promote the formation of a viscous chyme. After ingestion, salivary amylase should hydrolyze maltodextrin and solubilize the fiber, favoring the appearance of viscosity, which is called the amylase induced-viscosity system (amylase I-V). This system can attenuate postprandial glycemia [91].

Non-diabetic volunteers consumed bars containing 50 g of carbohydrates (control) and bars with 5.5 g of guar gum and 1.6 g of alginate (experimental). Compared to the control bar, glucose AUC was reduced by 33%, with a significant reduction at 15, 30, 45, and 120 min; the glycemia peak (45 min) was reduced by 30%, with suitable gastrointestinal tolerance within 24 h after ingestion [92].

Partially hydrolyzed guar gum (10 g) ingested by T2DM reduced HbA1c and a few parameters related to metabolic syndrome (waist circumference and serum trans fatty acids, for example) compared to baseline values after 4 and 6 weeks but did not change other biochemical parameters such as fasting glucose [32].

A test group consumed 7.5 g/day of dietary fiber per day for 12 weeks, with SDF (5.5 g/day) being a mixture of galactomannan, inulin, and β-glucan (in similar amounts), as well as a small amount (<10%) of glucomannan and alginic acid; insoluble DF (2.0 g/day), was mainly cellulose and with traces of hemicelluloses. Fasting plasma glucose decreased gradually over time, which was not observed in the placebo group. Similarly, Insulin and HOMA dropped in 12 weeks. When comparing the week 12 results between the test and placebo groups, only the reduction in fasting glucose was significant. These results indicate that fasting glucose and insulin resistance were reduced even with continued moderate dietary fiber intake, which was observed in men with mild hyperglycemia and visceral fat obesity [93].

The dose and form that guar gum administered can affect the flattening of plasma glucose curves. When 5 g of guar gum was added to bread, the glycemia peak was reduced by 41%, but with the same 5 g added to the soup, the reduction was 54% due to the complete hydration of the guar gum in the liquid matrix as opposed to the incomplete hydration of the bread. With 10 g of guar gum (5 g in bread + 5 g in soup), the reduction was 68%. The peak reduction in insulin was 37%, 50%, and 65% for 5 g of guar gum in bread, 5 g in soup, and 10 g, respectively. The difference between the 5 g dose in bread and the 10 g dose was significantly different for the reduction in blood glucose and insulin peaks. The results indicate that incorporating only 5 g of guar gum appears to be more effective if added to liquid foods [94]. However, juices with similar viscosity and enriched with a guar gum/xanthan gum (50:50) or konjac-mannan/xanthan gum (90:10) mixture to a control beverage, do not reduce postprandial glucose and insulin response when compared with the control, which could be due to the lower concentration of polysaccharides (below 1 g) [95].

A study by O’Connor and Campbell (2016) demonstrated that the two fiber composite products (a snack bar containing whole-grain high-amylose maize flour and guar gum and a smoothie-type beverage with guar gum, both ~15 g DF) reduced postprandial serum glucose and insulin responses compared to controls with maltodextrin (bar and drink ~4 g DF) in 240 min. The same occurred after the second meal, although this second reduction was more modest [96].

A meta-analysis that included 28 clinical trials (n = 1394, both genders, >47 years old), lasting from 3 to 52 weeks, evaluated the effects of guar gum, psyllium, konjac gum, β-glucan on markers of glycemic control in type 2 diabetes. Viscous fiber at an average dose of ∼13.1 g/day reduced HbA1c, fasting glucose, and HOMA-IR compared to control or standard treatment [97]. The authors considered this evidence to act moderately on these parameters but with a reduction in HbA1c that exceeded the limit established by the U.S. Food and Drug Administration (≥0.3%) to develop new antihyperglycemic drugs. They concluded that these results indicate that SDF can be an essential tool in the treatment of T2DM, but the authors reason that 13 of the included studies were less than 8 weeks in duration, and as HbA1c reflects blood glucose levels in the previous three months, the inclusion of shorter studies may have underestimated the effect due to their duration [97].

Some studies evaluated glycemic response with the ingestion of guar gum as a preload, alone or in conjunction with other foods, before the control food intake. For both whey (17 g) and whey (17 g)/guar (5 g) preloads, when consumed by T2DM volunteers 15 min before a mashed potato meal, reduced postprandial glycemia, which was associated with slowing gastric emptying. However, a low dose of guar gum (5 g) was less effective as a preload for glucose-lowering and did not slow gastric emptying [98]. A preload containing 16.4 g of whey protein and 4.4 g of guar was ingested by older subjects before consuming a glucose drink, and this was able to reduce plasma glucose. However, it stimulated plasma insulin and did not change gastric emptying in relation to the control. The authors considered that the glucose-lowering effect might be related to the delay in glucose absorption in the small intestine and the stimulation of insulin and not due to the deceleration of gastric emptying. In this study, gastric emptying was evaluated by scintigraphy. In the first study, a stable isotope breath test technique was used, which may have failed to assess gastric emptying and intestinal absorption adequately [99].

A preload with partially hydrolyzed guar gum (HG) plus highly cooked potato (HP) resulted in a better glycemic response when ingested before a rice meal (R) on the iso-carbohydrate basis, even compared to the suitable glycemic response arising from consuming HG or HP, both taken separately before co-ingestion of R + HP or R + HG, respectively. The authors hypothesize that incretin hormones can only effectively induce insulin secretion with an elevated blood glucose level. An appropriate combination of high-GI foods and dietary fiber preload could facilitate this release despite the reduced spike in blood glucose [100].

No significant differences were observed in plasma glucose of non-diabetic volunteers resulting from the consumption of reference bread and bread supplemented with guar gum at three levels (50, 100, and 150 g/kg of bread) at 60 min; however, there was a significant difference at 30 min with 100 g of added guar gum/kg. A significant difference in serum insulin was observed between the control and 50 and 150 g guar gum/kg bread at 30 min and with 50, 100, and 150 g of guar gum/kg at 60 min. Bread with 100 g of added guar gum/kg reduced the insulin response by 48% at 60 min and also achieved suitable acceptability [85]. Two different functional breads, with the addition of guar gum (2%) and inulin (8%) independently and in combination (guar gum 1% + inulin 4%), significantly decreased postprandial blood glucose and glycemic response of pita (GI of guar gum bread = was 55, GI of inulin = 57, GI of combined = 63) and Tandoori (GI of guar gum bread = was 57, GI of inulin bread = 60 GI of combined = 74), compared to the control bread (GI = 100). The authors explain that gastrointestinal movement changes with the ingestion of DF, delaying digestion and forming a thick physical barrier that hinders the absorption of carbohydrates. In addition, DF reduces the action of amylase to hydrolyze the starch granules and produces short-chain fatty acids resulting from fermentation [86]. Enzymatically or chemically modified guar gum leads to the reduction in molecular weight of native guar gum, presenting low viscosity in aqueous solutions, and its specific physicochemical properties make it possible to improve the quality of food products. This gum, when partially hydrolyzed, can be used for the development of fiber-enriched processed food products such as cookies, bread, noodles, yogurt, and others [14], but the lower viscosity can reduce postprandial blood glucose attenuation. However, the mechanisms involved in the physiological benefits of guar gum ingestion are not precisely known, and factors such as food matrix and food processing conditions may play a significant role [87].

### 3.7. Konjac Gum (Glucomannan)

Konjac glucomannan intake 30 min before performing the OGTT could lower the rise of blood glucose in comparison with the placebo; however, no statistical significance was seen for insulin. Long-term glucomannan supplementation significantly reduced the 120min glucose AUC of OGTT and HOMA-IR [88]. The intake of 4 g of konjac glucomannan added to noodles reduced the GI from 77 to 34, and volunteers with T2DM had significantly reduced HbA1c after 4 weeks of consumption of the supplemented noodle. This effect was not observed in individuals with metabolic syndrome, nor other glycemic effects (fasting blood glucose, insulin, HOMA-IR) for both groups [89]. A meta-analysis including six trials concluded that glucomannan supplementation reduced fasting blood glucose compared to the control groups, with a significant effect on diabetic patients but no effect on postprandial glucose of healthy volunteers [101].

### 3.8. Pectin

The effects of 10 g of pectin or cellulose on plasma glucose were studied in healthy women. The AUC of a diet with pectin was 122.7 mg/dL and with cellulose was 147.6 mg/dL, both were significantly lower than in comparison with the control diet (197.1 mg/dL), but there were no significant differences between dietary fiber type [102].

Data from six varieties of date palm fruits (*Phoenix dactylifera* L.) were classified as foods with a low GI (between 31 and 52), although these fruits are considered rich in carbohydrates. Their low GI values were attributed to the pectin content. The fruits of the variety that showed the lowest glycemic response were those that contained the highest sucrose content (~17%) and total sugars (~87–90%), and the content of pectin varied, curiously between 1.88% and 4.43% [103].

### 3.9. Pullulan

Pullulan is an extracellular polysaccharide excreted by the fungus *Aureobasidium pullulans*. In a crossover, double-masked, two-treatment study with juice-like beverages containing 50 g of carbohydrate from pullulan or maltodextrin control, incremental peak blood glucose concentration was significantly lower, and the iAUC glucose curve was reduced by 50% when subjects consumed pullulan [17]. A study with the same quantities of pullulan and beverages showed that at low molecular weight, pullulan did not reduce the incremental plasma glucose response compared to maltodextrin. However, the postprandial incremental serum insulin response was reduced by pullulan. The incremental peak and AUC insulin were significantly reduced by 23% and 20%, respectively, when subjects consumed pullulan compared to maltodextrin [104].

Seven drinks were tested: (1) Pullulan; (2) Pullulan + Soluble Corn Fiber (SCF) 70; (3) SCF 70; (4) Resistant starch (RS) 75; (5) RS 60; (6) RS + SCF 70; and (7) Soluble Fiber Dextrin (SFD) containing corn-based fiber ingredients. All evaluated fibers resulted in significantly lower glycemic and insulin responses for iAUC and at all time points compared to the control. Among the fibers, only the glucose iAUC and the iAUC insulin of the SFD produced values that were higher and different from the others. All of these new corn-based fibers produced lower postprandial glycemic and insulin responses than the control (25 g glucose) [40].

A study with long-chain pullulan (LCP) produced the lowest blood glucose excursions after 150 min, medium-chain pullulan (MCP) showed an intermediate standard, and maltodextrin (MD) was the highest. The smallest increase in blood insulin was observed for LCP, and the biggest rise was observed with MD. MCP showed a smaller insulin excursion than MDX, but this was not significantly different. AUC (150 min) for insulin was significantly lower for LCP compared with MDX [105].

### 3.10. Resistant Dextrins

A meta-analysis involving 37 randomized (n = 943), placebo-controlled studies assessed the glycemic response resulting from the ingestion of 3 to 10 g of resistant maltodextrin (RMD) with low viscosity [106]. The reduction in glycemic response was significant and dose-dependent, and no relationship was observed with the number of available carbohydrates ingested. The reduction in glycemic response compared to controls was at least 20% for every 10 g RMD ingested in the 37 studies. Although the intake of RMD consumed in beverages exhibited greater effectiveness, when the food was consumed in liquid form (10 studies), such as soups, porridges, and yogurts, there was no difference in relation to solid foods. Six studies also evaluated insulin response, which reduced an average of 25% with ingestion of 5–10 g of RMD compared to controls with foods containing 50 to 130 g of available carbohydrates. Although RMD is not very viscous, it can reduce the glycemic and insulin response when consumed with foods that are sources of available carbohydrates. The proposed mechanisms mainly include delayed gastric emptying; reduced nutrient absorption through faster intestinal transit; enzyme inhibition; and improved insulin response. Another advantage of RMD would be the low viscosity of this fiber, which reduces palatability issues and promotes its use in more products than viscous polysaccharides. The authors report that the results were independent of the amounts of carbohydrates, proteins, and fat available in the foods consumed and did not depend on the type of carbohydrate (mono or disaccharides or long-chain) [106].

Nutriose is a polysaccharide and a commercially soluble dextrin dietary fiber, with a mixture of polymers (GP~12 to 25), with high molecular weight. It is obtained from corn and wheat at high temperatures, with hydrolysis and repolymerization, generating bonds that are not found in starch and are resistant to enzymatic action, such as α-(1,6) or β-(1,6) linkage. A test group, which received 34 g/day of Nutriose for 12 weeks, exhibited a reduction in glucose (4%) and insulin (12%) in plasma, but it was not significant in relation to the control group that consumed maltodextrin. However, the HOMA-IR reduction was 18%. A significant reduction also occurred in relation to HbA1c and other parameters related to metabolic syndrome, which was more accentuated in volunteers with metabolic syndrome compared to non-carriers [20].

A study that evaluated parameters of oxidative stress in women with T2DM, including serum levels of glycemic indices, observed a reduction in fasting glucose among volunteers who ingested 10 g of Nutriose for 8 weeks in relation to the control group; however, there was no significant difference in HbA1c [107]. Glucose AUC after a morning drink (7 g of 14 g/day) was lower with Nutriose compared to the control on day 28. Moreover, for the Nutriose treatment, the mean AUC was lower on days 14 and 28 compared to day 1. These differences were not found in the overweight group, only in normal-weight volunteers. Postprandial insulin was unaltered by supplementation [19]. However, a review evaluated the results of six studies (n = 6 to 22 volunteers, with normal body mass index or overweight with Nutriose (~50 g), resistant dextrin containing 85% resistant starch). The glucose iAUC observed was low, ranging from 25% to 48%, and the insulin responses were from 13% to 20%, indicating that the ingestion of this product causes a reduction in these responses [108].

When the substitution of 15% and 30% tapioca-resistant maltodextrin (TRM), a new non-viscous soluble resistant starch, was tested in oral nutritional supplements in an acute study, it was observed that the postprandial plasma peak of glucose and insulin in 30 min of TRM30 was lower compared after intake of TRM15 and original formula. Replacement of TRM by 30% and 15% reduced incremental peak plasma glucose by 9.50% and 10.93%, respectively. However, those reductions were not significant. iAUC insulin was lower compared to TRM15 and the original formula, but there was no significant difference for iAUC of plasma glucose. Long-term (56 g/12 weeks) use of TRM30 resulted in a reduction in HbA1c in prediabetes and normoglycemic participants [109]. However, in another acute study, at 30 min after intake of drinks, glycemia of TRM was significantly lowest compared with glucose, tapioca maltodextrin, MIX15% (7.5 g TRM + 42.5 TM), and MIX50% (25 g TRM + 25 TM) drinks. In addition, mean plasma glucose concentrations after the consumption of TRM were lower when compared with the GL treatment at 60 min [110].

Soluble corn fiber (SCF) is obtained by isolating an oligosaccharide-rich fraction from partially hydrolyzed corn syrup. The position of the glycosidic bonds present reduces SCF digestibility. The glycemia peak after breakfast, replacing 30% of the available carbohydrates in a normal diet with PDX or SCF (~57 g PDX and 55 g SCF per day) was significantly lower with both diets compared to the consumption of the full diet; however, after the second meal, only the SCF diet reduced the glycemia peak. After both breakfast and lunch, the insulin response (AUC) was significantly lower after consumption of the SCF or PDX diets than the full diet. PDX also had a pronounced suppressive effect on appetite assessments [41]. Therefore, the use of SCF as a replacement for carbohydrates in meals (25–55 g) can reduce peak glucose and insulin as well as glucose and insulin iAUC compared to controls, displaying its benefits in controlling the postprandial glycemic response.

Volunteers treated with soluble corn fiber (SCF)-replacing 50% of total carbohydrate with SCF in four meals (two solids and two drinks), had significantly lower glucose iAUC 130 min and insulin iAUC 130 min compared to the controls, and the intake of foods with maltodextrin (MD) were not significantly different from glucose. At each point, rice with SCF was also lower than glucose; however, in the case of drink, it was better between 55 and 100 min. The insulin response was lower between 40 and 130 min for the drink and up to 70 min for rice with SCF. The MD drink significantly increased postprandial glucose and insulin responses by 20% and 40%, respectively, compared to the SCF drink. The authors attributed these effects to the ability of SCF to resist digestion and absorption in the small intestine and undergo fermentation in the large intestine [111]. According to Kendal et al. (2008), only 14.5% of SCF is digested in an in vitro assay, and short-chain fatty acids produced by colonic fermentation can alter intestinal motility by acting on intestinal endocrine cells and/or neurons of the enteric nervous system [42].

### 3.11. Xanthan Gum

The GI of the biscuits with added 10 g of viscous soluble fiber (70% glucomannan + 30% xanthan) was 26 (16–36) and 37 (27–47) for healthy and diabetes mellitus participants, respectively. These values were significantly lower than for white bread (GI = 100), white bread with 12 g of margarine, and control biscuits, both in healthy participants (GI = 108 and GI = 101, respectively) and in participants with T2DM (GI = 103 and GI = 94, respectively) [112]. A systematic review with 14 studies evaluated interventions with either xanthan gum, pullulan, or dextran, with a wide variation in the amount of hydrocolloid supplementation provided and methods of preparation. Higher intake levels and longer-chain hydrocolloids promoted reduced postprandial blood glucose responses in half of the studies. A significant reduction in postprandial blood glucose was observed when xanthan gum was added to the cooking process of muffins and rice [113].

### 3.12. Soluble Dietary Fibers (Various)

A systematic review and meta-analysis included 12 clinical trials (n = 609 overweight or obese participants) with a duration of 2 to 17 weeks. The participants consumed varying doses of soluble DF supplements (3 to 34 g/day of fiber non-viscous (manno-oligosaccharides, galacto-oligosaccharides, and fructo-oligosaccharides), and viscous (β-glucan, flaxseed mucilage, mannans, pectins, oligofructose, wheat, and maize-derived dextrin), or a mixture of both types). SDF supplementation reduced fasting glucose compared to the effects of placebo treatments, and the dose and duration of intervention did not affect the results. Meta-regressions revealed differences in HOMA-IR by fiber type, with a more pronounced reduction in resistance to insulin observed with non-viscous fermentable fiber. The authors caution that there is considerable heterogeneity between studies, so the results should be viewed with caution; nevertheless, the results suggest that soluble fiber supplementation alone may contribute to the homeostasis of glucose and insulin, as well as weight control [114].

A study tested ice cream formulations with soy milk and added glucomannan flour (0.5%, 1.5%, and 2.5%). The one with 2.5% glucomannan flour (2.75% SDF and 1.24% IDF) and 81.5% soy milk were the preferred ice creams of volunteers, based on the organoleptic analysis and presented the GI = 51 and GL = 9, while a standard formulation (1.56% SDF and 0.56% IDF) showed GI = 76 and GL = 12. Glucomannan flour has a high fiber content (5.9 g/100 g) which could present a physical obstacle in the digestion process and affect the GI value [115].

Table S1 (Supplementary Material) provides a summary of the positive effects observed in the human studies mentioned above.

## 4. Glycemic Response to Bread

Bread is one of the most studied foods involving glycemic response. Its spongy structure and highly gelatinized starch make it easily accessible to amylase, which causes high glycemic responses [116], and it is even used as a reference food for the determination of GI in many studies. The desired reduction in GI in bread can be achieved by making products with a more compact structure or greater density. Thus, production must involve reducing the kneading time or using less yeast to make the structure less porous, generating more dense products, which would alter the hydrolysis process in the mouth and the gastrointestinal transit. However, consuming bread with these characteristics involves changes in the eating habits of consumers, who would need to consider the change in texture as a physiological benefit [9].

The selection of raw materials can also be an essential factor in reducing GI. Studies with cereal-based products demonstrate that the preservation of structures during digestion seems to be a more important factor than the degree of starch crystallinity or the presence of SDF [9].

Viscous fibers are types of hydrocolloids that have been used in gluten-free products, mainly in bread, and this includes the fibers mentioned in this review, such as xanthan, konjac, guar gum, psyllium, pectin, and alginate, among others, that promote interactions between flour and other ingredients. They increase viscosity and air incorporation and can improve dough handling, baking quality, and texture, in addition to affecting the glycemic index (GI) of the final product and increasing the dietary fiber content of gluten-free baked goods. Many studies have reported these benefits and demonstrated that viscous fibers are important both from a physiological and technological point of view [117]. The addition of functional ingredients can increase satiety levels, sensory characteristics, and shelf life, and ingredients such as inulin and guar gum can be used to contribute to reducing the glycemic response [86].

At the same time that several studies have demonstrated the potential of soluble fiber in the lowering of glycemic response, the same does not usually occur with studies involving only TDF. Bread enriched with certain types of TDF did not show suitable potential to reduce the glycemic response when subjects with type 2 diabetes consumed foods (bread + breakfast cereal) with high addition of ground ultrafine wheat bran (19 g/day) for three months and products with low addition of this source of DF (4 g/day of cereal fiber), in the control phase [118]. Similarly, the consumption of whole-grain products with 112 g fiber/day for six weeks made with ground whole wheat did not alter insulin sensitivity, fasting blood glucose, or markers of lipid peroxidation and inflammation in overweight individuals [119]. Predominant consumption of bran or more finely ground wheat in two studies may have reduced the expected effects on glucose and insulin, as wheat contains less soluble and more insoluble fiber than rye, oats, or barley.

The presence of intact structures not accessible to human amylases, as well as a low pH, can delay gastric emptying or create a barrier to starch digestion. The incorporation of extracted cereal fibers, the addition of legume fractions, or viscous or non-viscous fibers may also be effective. Adding intact or partially ground barley grains was demonstrated to be effective in reducing the glycemic response. However, the effect of whole wheat flour is less evident unless varieties with a high β-glucan content are used [116].

## 5. Technological Aspects and Challenges

The addition of SDF to foods may be restricted by its effects on the product’s palatability. However, the beneficial properties of SDF, such as viscosity, can be altered by food processing to improve palatability. Nevertheless, the alteration of physicochemical properties by heating can lead to depolymerization and thus alter the effectiveness of SDF on the glycemic response. In this sense, the health claim by EFSA (2011) [120] about β-glucan refers to that is naturally present or added to foods. However, they warn that viscosity depends on the raw material, molecular weight, β-glucan dissolution, and processing applied.

When β-glucan barley is used as a source of fiber added to wheat flour, fractions obtained by the freeze-drying technique can be applied, as it does not negatively affect the sensory characteristics of bread. However, the β-glucan enrichment technique from water extraction appears to be more suitable since it presented sensory characteristics in bread similar to those produced with wheat flour [67].

According to Cassidy et al. (2018) [27], alginate only becomes viscous after reaching the acidic environment of the stomach, which can improve its palatability. Several factors can interfere with the gel strength, such as marine algae species, molecular chain lengths, mannuronic/guluronic acid ratio, quantity, and food matrix used. Thus, all these factors must be considered when formulating functional products that aim to reduce the increase in postprandial glycemic response.

Studies with guar gum and corn flour with a high amylose content avoided heat treatment so that there would be no physicochemical alterations in the fibers [96]. Nevertheless, studies with processed products are necessary to assess how the degree of processing can compromise the use of these fibers. These effects have already been observed when enrichment with guar gum is employed as a DF source in quantities necessary to promote physiological effects, which changed the palatability of the products, possibly limiting the application of this fiber source in industrialized products [90].

Similarly, the use of powdered gray mushroom, *Pleurotus ostreatus*, in formulation with 8% concentration did not significantly alter the sensory properties and presented low GI. In comparison, at 12% concentration, it produced biscuits with greater firmness, stronger aroma, and darker color, which may have resulted in lower markers during the sensory evaluation [81]. Thus, various concentrations and formulations/processes must be tested to obtain the best cost/benefit involving significant physiological effects and sensory characteristics.

Soluble corn fiber (SCF) added to glutinous rice and steam-heated for 5 min showed an accentuated reduction in glycemic and insulin response compared to glucose beverages and meals comprising maltodextrin in glutinous rice or drink and a drink added with SCF [120] Nevertheless, the authors note that the gastric emptying rates for solid foods are slower compared to liquid foods, which can affect both glycemic and postprandial insulin. Replacement of part of the available carbohydrates by foods produced with SCF in the formulation (breakfast cereals, pudding, and two powder mixes for instant drinks) also promoted the reduction of postprandial glycemic peaks, including after the second meal [28]. These two studies showed that the SCF added to both solid and liquid meals was effective. However, it can have a different impact, depending on the type of food used as a vehicle.

An increase in protein, amylose, and dietary fiber concentration can be obtained by using a vertical stone milling process to produce stone-milled wheat bran powder. This product favors the formation of the starch-protein complex, which reduces starch digestibility and thus might reduce the glycemic response [121].

When fresh noodles were partially substituted with banana flour (BF), from 0% to 40%, the viscoelastic properties increased, while tensile strength and elasticity decreased. Estimated GI was reduced from 77 to 63 (intermediate GI), while RS content increased from 5.56% to 23.31% (very high RS). Furthermore, the effects of xanthan gum (XG), guar gum (GG), and carboxymethyl cellulose (CMC) individually at 1.0% and 1.5% levels on the quality of dried noodles substituted with 30% BF (DBF30) were investigated. DBF30 with XG had the shortest rehydration time (6.5 min), while DBF30 with CMC had the longest rehydration (8.5 min). The added hydrocolloids increased rehydration and decreased the cooking loss of DBF30 while also increasing the tensile strength and elasticity of DBF30. Furthermore, the hydrocolloids increased RS content and reduced the GI of DBF30 in vitro. These results reveal that adding BF and hydrocolloids to noodle products can provide high nutritional quality with enhanced quality characteristics [122].

Resistant maltodextrin can reduce the glycemic response despite its low viscosity, which reduces palatability issues, and this facilitates its use in more products than other more viscous polysaccharides [106].

The limitation in the use of viscous SDF due to viscosity and physicochemical properties can be solved by adding DF mixtures, which have already been observed with SDF mixtures of glucomannan, sodium alginate, and xanthan gum [27].

## 6. General Considerations

Extensive research has investigated β-glucans and their positive effects in reducing the postprandial glycemic response. However, other SDFs (guar gum, psyllium, composite fibers, etc.) have achieved promising results, with some showing greater efficacy than others.

The gastric emptying rate can affect postprandial blood glucose and insulin, so solid foods, especially with large, hard, or dense particles, tend to have slower gastric emptying compared to liquid foods or those with smaller particles. However, adding guar gum was found to be more effective in reducing the glycemic response when added to liquid food [94].

The viscosity of some soluble fibers has been reported as the property responsible for reducing the glycemic response of these fibers, which was not demonstrated in a study with β-glucan on the glycemic response with the change in viscosity through volume. In the study by Kwong, Wolever, Brummer, and Tosh (2013) [69], the addition of β-glucan and its molecular weight was the most critical factor in decreasing the glycemic response.

Some soluble fibers used in the studies presented here do not have high viscosity. However, even with low viscosity, the high molecular weight or size of the pullulan chain contributed to the reduction of the glycemic response [17,104]. Concerning resistant dextrin, Livesey and Tagami (2009) [106] hypothesized the reduction in glycemia due to factors such as slower gastric emptying, movement of chyme to distal sites in the intestine where absorption may be slower, enzymatic inhibition or also degradation, oxidation or browning of RMD during cooking, but there seems to be a dose–response effect and more pronounced if consumed in beverages.

The most used markers to evaluate the glycemic response are glucose and insulin levels, but in longer studies, HbA1c has also been considered. However, it is essential to appraise the nature and limitations of HbA1c as a marker. It depends on the interaction between the lifespan of the erythrocyte and the concentration of blood glucose. As the mean erythrocyte lifespan is approximately 120 days (17 weeks), HbA1c reflects the average glucose concentration during the preceding 8–12 weeks, so studies with less than 12 weeks are in the range of underestimating the effects of the intervention [97]. Antidiabetic medication has its full effect on HbA1c after 20 weeks [123].

The degree of starch crystallinity or SDF’s presence contributes to the reduction in glycemic response. However, the preservation of structures in cereal products during digestion seems to be a significant factor [9]. Wheat and its finely ground bran do not seem to inhibit a rapid glycemic and insulin response since this cereal has less soluble fiber than other cereals. The spongy structure of bread, with much gelatinized starch, makes bread easily digested by human digestive enzymes. At the same time, production involving less dough growth, with less kneading and less yeast, would make bread more compact, with a firmer structure, making the food less sensitive to enzymatic action with a consequent reduction in the glycemic response [118,119].

Besides viscosity and molecular weight, the hydration properties, particularly water holding capacity, the amount of bound water, and water mobility, were recently pointed out as worthy of investigation for the characterization of soluble dietary fibers and their impact on glucose release. The study investigated five different types of SDF using an in vitro digestion model, and a strong correlation between hydration properties and glucose diffusion and release was determined [124].

SCF consumed with food or substituting part of the carbohydrates in products has effectively reduced the glycemic and insulin response without changing the viscosity resulting from the processing.

A large part of the studies was carried out with bread and drinks. It is crucial to test the addition of different sources of soluble dietary fiber in different food matrices since there may be nutrient complexity and/or interference with nutrient absorption by the presence of some components, such as proteins and lipids.

Of the 64 studies surveyed in this review, 67% were carried out with healthy, non-diabetic adults and 8% with adults with T2DM. Only one was performed with an elderly (healthy) cohort and one with adolescent T1DM. After evaluating the safety and efficacy of a healthy public, studies should be carried out with volunteers with diseases that may benefit from the impact of SDF on the glycemic and insulin response, as well as studying this benefit in long-term studies.

It is important to remember that some types of fibers declared as components of food products are the subject of health claims already provided by the European Union, for example [125]. In most cases, the claims were aimed at intestinal function and cholesterol control. European Union documents of Commission Regulation (EU) n^o^. 432/2012 (16 May 2012) [125] show that some types of dietary fiber or non-digestible carbohydrates, used as a whole or to replace part of the soluble sugars with the aim of reaching the amount to be used on the label in the information “reduced in sugars”, may contribute to the reduction of the postprandial glycemic response of foods. However, when responding to a question about why 16 applications for authorization to use the health claim were not approved, the European Food Safety Authority (EFSA) reported that it does not consider low-GI carbohydrates to be sufficiently characterized yet [126]. More studies with different sources and types of SDF are essential to provide scientific evidence for health or functional claims for dietary fiber and reduced glycemic response.

Although the number of studies and results are varied, the viscosity of SDF has generally been shown to reduce glycemic and insulin responses efficiently; nevertheless, some technological challenges still require further study to avoid interfering with important metabolic responses in the human body.

## Supplementary Materials

The following supporting information can be downloaded at: https://www.mdpi.com/article/10.3390/foods11233934/s1. Table S1. Characteristics of clinical studies with significant responses on glycemic and insulin response.

## Abbreviations

AUC = area under the curve, iAUC = incremental area under the curve, AX = arabinolxylan, DF = dietary fiber, EFSA = European Food Safety Authority, GI = glycemic index, GL = glycemic load, HbA1c = glycated hemoglobin, HOMA = homeostasis model assessment, II = insulinemic index, KGM = Konjac galactomannan, OBG = Oat β-glucan, OGTT = oral glucose tolerance test, PBGR = peak blood glucose rise, RMD = resistant maltodextrin, SCF = soluble corn fiber, SDF = soluble dietary fiber, TAC = total available carbohydrates, TDF = total dietary fiber, TD2M = type 2 diabetes mellitus, TMD = tapioca-resistant maltodextrin.
