# Supplementary material for: The Effects of Soluble Dietary Fibers on Glycemic Response: An Overview and Futures Perspectives
Source: Foods. 2022 Dec 6;11(23):3934. doi: 10.3390/foods11233934 (PMC9736284; doi:10.3390/foods11233934)
Supplement: Supplementary file 1 [file foods-11-03934-s001.zip › foods-2010671-supplementary.pdf]

Table S1. Characteristics of clinical studies with significant responses on glycemic and insulin response

Type of fiber	Control	Volunteers (number/health status or age)	Dose	Effects on glucose with significant difference	Effects on insulin with significant difference	Publication
Alginate	Meal without additive	7 T2DM	5.0 g	Reduced AUC = 31%d	Reduced AUC = 42%	Torsdottir et al. 1991 [37]
Alginate	Acidic-gelling control	40 healthy - lower body fat group (< 16.10%); upper body fat group-(≥ 16.10%)	1.5 g	Reduced peak = 14%<br>Reduced AUC = 52%<br>No interference from body composition	Not evaluated	Harden et al. 2012 [35]
Alginate	Low volume control - 330 mL<br>High volume control - 500 mL	20 healthy	Low volume - 9.9 g<br>High volume - 15 g	iAUC High volume -alginate vs High volume control = reduction 40%	No significant effect	Jensen et al., 2012 [36]
Alginate	Chocolate milk (CM) – control 1<br><br>Water – control 2	24 healthy	1.25%<br>2.5%<br><br>2.5%	Reduced peak CM 2.5% vs CM 1.25% = 32%<br>Reduced peak CM 2.5% vs CM = 46%<br>3 treatments with alginate - reduced postprandial blood glucose in 0–120 min and 120–260 min	Reduced peak CM 2.5% vs CM = 46%<br>Reduced CM 2.5% ~ 2.5% solution (control 2)	El Khoury et al., 2014 [34]
Alginate (Alg)	Control meal – noodles (CM)	15 healthy	3.2 g and 5.0 g	Reduced peak Alg 5% vs CM =-11%; Alg 8% vs CM=15%<br>Reduced AUC Alg 5% vs CM 15%; Alg 8% vs CM=21%	Not evaluated	Kato et al., 2018 [38]
Alginate (Alg)+ Soy protein isolate (SPI), pH 6 and 7	Beverage sugar (C)	12 healthy	0,63 g	Reduced peak SPI6, SPI7, SPI6+Alg, SPI7+Alg vs C=<br>33.4%, 36.3%, 53.2%, 58.5%<br>Reduced glucose peak<br>SPI6, SPI7 vs SPI6+Alg, SPI7+Alg<br>Reduced Glucose AUC<br>SPI6, SPI7, SPI6+Alg, SPI7+Alg vs C	Reduced peak insulin SPI6, SPI7, SPI6+Alg, SPI7+Alg vs C	Huang et al., 2019 [39]
Arabinoxylan (AX)	Bread	14 healthy	6 g AX	Reduced peak (6 g) vs control (mmol/L) = 6.3 vs 7.2	Reduced AUC = 17.0%	Lu et al., 2000 [40]

Table 2. Characteristics of clinical studies with significant responses on glycemic and insulin response

			12 g AX	Reduced AUC = 20.2%<br>Reduced peak (12 g) vs control (mmol/L) = 5.9 vs 7.2<br>Reduced AUC = 41.4%	Reduced AUC = 32.7%	
Arabinoxylan	Liquid meal challenge test	12 overweight and impaired glucose tolerance	15 g (6 weeks)	Reduced postprandial response (in 240 min) after 6 weeks<br>Reduced fasting glucose (mmol/L) = 6.3 vs 5.4	Reduced postprandial response (in 240 min) after 6 weeks	Garcia et al., 2006 [44]; Garcia et al., 2007 [43]
Arabinoxylan	Bread and muffins	15 with DM2 and overweight	15.1 g/day (5 weeks)	Reduced fasting glucose (mmol/L) =7.6 ± 0.3 to 7.0 ± 0.3<br>Reduced OGTT (mmol/L) =15.2 ± 0.9 to 13.4 ± 0.9	Reduced fasting insulin (pmol/L) = 98 ± 10 to 77 ± 6<br>Reduced OGTT (pmol/L) = 656 ± 174 to 493 ± 121	Lu et al., 2004 [41]
Arabinoxylan (AX)<br>β-glucan (BG)<br>Rye kernels (RK)	Wheat bread	15 with metabolic syndrome	Bread AX – 7.1 g (GI=89)<br>Bread BG – 2.6 g (GI=84)<br>Bread RK – 6.1 g (GI=77)	Peak glucose AX < control<br>iAUC BG vs control (mmol/L) = 266 ± 27 vs 330 ± 36<br>iAUC RK vs control (mmol/L) = 249 ± 27 vs 330 ± 36	Reduced iAUC (nmol/L)<br>BG vs control =21.2 vs 25.3<br>RK vs control = 12.8 vs 25.3	Hartvigsen et al., 2014 [51]
Arabinoxylan (AX)<br>Rye kernels (RK)	Semolina porridge (0.9 g AX)	15 with metabolic syndrome	AX=3.5 g<br>RK=4.7 g AX<br>AXRK=4.4 g AX	iAUC AXRK vs control (mmol/L) = 201 vs 258<br>Peak glucose AXRK vs control (mmol/L) = 8.7 vs 9.9<br>Reduced iAUC_AX relative to control (80%)	iAUC AXRK vs control (nmol/L) =16.8 vs 22.5<br>iAUC RK vs control (nmol/L) = 13.9 vs 22.5<br>iAUC AX vs RK and AXRK (nmol/L) = 20.9 vs 13.9 and 16.8	Hartvigsen et al., 2014 [52]
Arabinoxylan	Breakfast	15 healthy	AX=6 g	No significant effect	iAUC (mmol × min/L) = 13.1 ± 2.56 vs 19.2 ± 4.9	Möhlig et al., 2005 [42]
Psyllium<br>(AX)	Placebo	37 TD2M	6.8 g/day<br>13.6 g/day<br>(12 weeks)	13.6 g and 6.6 g vs placebo<br>Reduced fasting glucose (4,8, 12 weeks)<br>13.6 g vs placeb<br>Reduced HbA1c (8 weeks)<br>13.6 g and 6.6 g vs placebo<br>Reduced HbA1c (12 weeks)	Not evaluated	Feinglos et al., 2013

Table 2. Characteristics of clinical studies with significant responses on glycemic and insulin response

Psyllium (AX)	Regular diet (control group)	40 TD2M	10.5 g/day 8 weeks	Intervention vs control group Reduced fasting blood sugar (mg/dl) = 163 to 119 Reduced HbA1c% = 8.5 to 7.5	Reduced insulin 27.9 to 19.7 µIU/mL Reduced HOMA.IR (11.3 to 5.8) and HOMA-β % (103 to 141)	Abutair et al., 2016 [46]
Psyllium (AX)	White bread (WB) Gluten free bread (GFB) 25 g available carbohydrates	13 healthy	17.14 g psyllium P-GFB	Reduced GI (25%) and GL (39%) P-GFB vs GFB and WB	Not evaluated	Fratelli et al., 2018 [49]
Psyllium (AX)	Cookies placebo	51 TD2M	10 g psyllium 12 weeks	Reduced fasting plasma glucose (mg/dl) Psyllium vs Placebo (151.5 ±43.1) vs (163.9 ± 39.5) and HbA1c % (7.6 ± 2.0) vs (9.0 ± 2.0) Reduced fasting plasma glucose (mg/dl) Psyllium – after 12 weeks (151.5 ±43.1) vs baseline (171.0 ± 39.1)	No significant effect	Soltanian et al., 2018 [48]
Psyllium (AX) Flaxseed	Cookies placebo	77 TD2M	10 g psyllium or 10 g flaxseed 12 weeks	Reduced fasting plasma glucose (mg/dl) Flaxseed and psyllium After 12 weeks (147.3 ± 43.0) vs baseline (167.0 ± 38.2) After follow-up 4 weeks (152.4 ± 38.0) vs baseline (167.0 ± 38.2)	No significant effect	Soltanian; Janghorbani, 2019 [49]
Psyllium (P)+ Chickpea flour (CF)	Rice flour () (75%) + cassava flour (25%) (GFB)	12 healthy	5,5% psyllium	Reduced GI CF (79) and CF+P (75) vs GFB (100) Reduced GL: 6.3 and 5.9 vs 12.4 Reduced iAUC mmol/L x min): 104 and 96 vs 134 Reduced glycemic response in 30, 45 and 60 min	Not evaluated	Santos et al., 2021 [50]

Table 2. Characteristics of clinical studies with significant responses on glycemic and insulin response

β-glucan<br>(barley PW)	Wheat bread<br>(GI=100;<br>II=100)<br>Bread +<br>common barley<br>(GI=83; II=81)	10 healthy	35% PW<br>50% PW<br>75% PW	Bread 35% PW – GI = 75<br>Bread 50% PW – GI = 65<br>Bread 75% PW – GI = 55	II = 74<br>II = 65<br>II = 68	Ostman et al., 2006 [54]
β-glucan<br>(oat and wheat)	Wheat bread	9 healthy	Raw rolled oats<br>Porridge - rolled oats<br>Porridge - oat kernels<br>Porridge - wheat kernels<br>Servings of 50 g of available carbohydrates	 <br>Porridge - oat kernel - GI = 60<br>Porridge - wheat kernel - GI = 67	 <br>II = 68<br>II = 68	Granfeldt et al., 1995 [55]
β-glucan<br>(BF-barley flour<br>SF- sieved fraction<br>WF-water-extract fraction)	Bread wheat flour	8 healthy	Bread 50% BF (2.4%)<br>Bread 50% SF (4.2%)<br>Bread 20% WF (6.7%)	Reduced GI - only WF<br><br>Bread 20% WF – GI = 72	Not evaluated	Cavallero et al. (2002) [56]
β-glucan	Granola - 0.6 g β-glucan<br>- in 40 and 60 g total available carbohydrate (TAC)	12 healthy	6.2 g/40 g TAC<br>6.2 g/60 g TAC<br>β-glucan<br>with High, Medium and Low MW (molecular weight)	Reduced peak = 40 g TAC > 60 g TAC<br>iAUC reduction = 40 g TAC > 60 g TAC<br>Peak and iAUC reduction = HMW 60 g TAC > MMW, LMW, and control;<br>Reduced peak = HMW 40 g TAC > MMW, LMW 40 g TAC and control<br>iAUC reduction = HMW ~ MMW 40 g TAC > LMW ~ control	Not evaluated	Regand et al., 2011 [57]
β-glucan	50 g glucose (250 and 600 mL)	15 healthy	4 g High MW<br>4 g Low MW	Reduced peak HMW vs peak LMW = 3.13 vs 3.82 mmol/L	Not evaluated	Kwong et al., 2013 [58]
β-glucan<br>(by oat-bran preload<br>- 5 min before)	White bread<br>(50 g available carbohydrate)	10 healthy	0.9 g (in 4.5 g oat-bran)<br>2.6 g (in 13.6 g oat-bran)<br>5.3 g (in 27.3 g oat-bran)	Reduced blood glucose<br>15 min – 27.3 g < 4.5 g and 0<br>30 min – 27.3 g < 13.6 g < 4.5 g and 0<br>45 min - 27.3 g < 13.6 g, 4.5 g and 0 g<br>AUC 27.3 g < AUC white bread	Not evaluated	Steiner et al., 2016 [63]

Table 2. Characteristics of clinical studies with significant responses on glycemic and insulin response

β-glucan	Breakfast with β-glucan (control)	33 normal -weight	4 g HMW	Reduced blood glucose – 30 min<br>Breakfast HMW vs control	Reduced plasma insulin at 30 and 60 min<br>Breakfast HMW vs control<br>Reduced AUC (μU/mL x 90 min)<br>1682 vs 2491	Zaremba et al., 2018 [59]
β-glucan	Cream of Rice (CR)	28 without diabetes	Oatmeal 2 g β-glucan (2gOBG), Oatmeal 4 g β-glucan (4gOBG), Oat-bran plus β-glucanase (4gloMW)	Reduced AUC 2 h<br>2gOBG, 4gOBG and 4gloMW vs CR<br>4gOBG vs 2gOBG and 4gloMW<br>Reduced AUC 3 h<br>4gOBG vs CR<br>Reduced glucose peak<br>2gOBG, 4gOBG and 4gloMW vs CR<br>4gOBG vs 2gOBG and 4gloMW	Reduced AUC 2h<br>4gloMW vs CR<br>Reduced peak<br>4gloMW vs CR, 2gOBG and 4gOBG	Wolever et al., 2020 [64]
β-glucan	Standard diet program (for 1 week)	T1DM adolescents	β-glucan 6 g/day (O6) for 1 week<br><br>β-glucan 3 g/day (O3) for 1 week	Reduced premeal and post meal blood glucose levels, and peak blood glucose in breakfast, lunch, and overall.<br>Delayed peaks for all time-points<br>O6 vs O3	Not evaluated	Bozbulut et al., 2020 [62]
β-glucan	50 g glucose (200 mL)	16 healthy	4 g oat β-glucan with MW and viscosity variables	Reduced iAUC 0–45 min < 63 vs 80 mmol × min/L (control)	Not evaluated	Wolever et al., 2020 [60]
β-glucan	OGTT	14 healthy	Cereal β -glucan<br>Low: 0.8 g; Medium: 3.2 g; High: 6.6 g<br>In evening meal / 3 days	Reduced blood glucose<br>Medium vs Low, High and baseline	Reduced blood glucose<br>Medium vs Low, High and baseline<br>HOMA-IR no difference	Telle-Hansen et al., 2022 [66]
β-glucan (BG)<br>Psyllium (husk)	Durum wheat semolina pasta	10 healthy	15% Barley Balance (BB) (25% BG)<br>15% psyllium<br>7.5% BB + 7.5% psyllium	BB vs control – GI = 33 vs 52<br>BB + psyllium vs control – GI = 35 vs 52	Not evaluated	Peressini et al., 2020 [65]

Table 2. Characteristics of clinical studies with significant responses on glycemic and insulin response

Pleurotus sajor-caju (PSC)<br>(BG source)	Biscuit without addition (3.37 DF; β-glucan (BG)=0.12%)	11 healthy	6.19% (BG=1.06%)<br>8.62% (BG=1.29%)<br>9.84% (BG=1.78%)	Reduced iAUC (mmol × min/L) = 8% (82.3 ± 11.0) and 12%, (79.7 ± 7.4) vs 0 (96.1 ± 8.1) and 4% (87.3 ± 10.4)<br>GI 4% = 52<br>GI 8% = 49<br>GI 12% = 47	Not evaluated	Ng et al., 2017 [70]
Pleurotus ostreatus (P.o) and *Pleurotus cystidiosus* (P.c)<br>(BG source)	Glucose (75 g)	22 healthy<br>28 T2DM on a controlled diet	50 mg/kg body weight	Reduced fasting glucose (healthy) (mmol/L) = P.o (4.5 to 4.3) and P.c (4.4 to 4.1)<br>Reduced postprandial (healthy) (mmol/L) = P.o 5.5 to 4.6) and P. c (5.3 to 4.6)<br>Reduced postprandial (T2DM) = P.o 14.9% and P.c 16.6%	Not evaluated for healty<br>Increased postprandial (T2DM)<br>P.o.(21.7%) and P.c (21.0%)	Jayasuriya et al., 2015 [69]
Pleurotus spp (oyster mushroom)<br>(BG source)	Ajwain biscuits<br>Time zero (T0)	120 T2DM	Ajwain + mushroom biscuits<br>Mushroom biscuits<br><br>DF dose not informed (for 3 months)	Reduced fasting glucose (mg/dL):<br>Ajwain + mushroom group (113.83 ± 4.03) vs T0 (225.41 ± 3.35)<br>Ajwain + mushroom (113.83±4.02) vs Ajwain group (310.33±3.31)<br>Mushroom group (112 ± 1.37) vs T0 (212.9 ± 4.29)<br>Mushroom (112 ± 1.37) vs Ajwain group (310.33±3.31)<br><br>Reduced HbA1c %:<br>Ajwain + mushroom group (7.27 ± 0.14) vs T0 (8.47 ± 0.17)<br>Ajwain + mushroom (7.27 ± 0.14) vs Ajwain group (9.98±0.14)<br>Mushroom group (6.99 ± 0.12) vs T0 (8.00 ± 0.13)<br>Mushroom (6.99 ± 0.12) vs Ajwain group (9.98±0.14)	Not evaluated	Agrawal et al., 2010 [68]
Pleurotus ostreatus<br>(BG source)	Time zero	27 men with T2DM and hypertension	3 g/day (for 3 months)	Reduced fasting glucose (mmol/L) = 10.36 ± 0.72 to 8.48 ± 0.58	Not evaluated	Choudhury et al., 2013 [71]

Table 2. Characteristics of clinical studies with significant responses on glycemic and insulin response

				Reduced HbA1c% = 8.2 ± 0.36 to 7.1 ± 0.31		
Pleurotus sajorcaju powder (PSP) (BG source)	Tortilla control (T0%) Glucose	14 healthy	Partial replace - Tortillas with 5, 10 and 15% PSP	Reduced iAUC (mmol x min/L): T15% (119 ± 17) and T0% (106 ± 11) vs Glucose = (201 ± 9) Reduced GI: T15% (53) and T0% (58) vs Glucose = (100)	Not evaluated	Ishak et al., 2020 [73]
Pleurotus eryngii (P.e)	Control meal	19 with unhealthy obesity	Meal + P.e	Reduced iAUC (mg/dL X 180 min) Meal + P.e (948) vs Control meal (2160)	No significant effect	Kleftaki et al., 2022 [72]
Guar gum (GG)	Bread<br>Soup<br>Bread + soup	5 healthy	5 g + bread<br>5 g + soup<br>10 g + bread and soup	Reduced peak = 41%<br>Reduced peak = 54%<br>Reduced peak = 68%	Reduced peak = 37%<br>Reduced peak = 50%<br>Reduced peak = 65%	Wolever et al., 1979 [78]
Guar gum	Bread	11 healthy	5% bread weight<br>10%<br>15%	10% reduction in 30 min	5% -30/60 min = reduction<br>10% - 60 min = reduction (48%)<br>15% - 30/60 min = reduction	Ellis et al., 1981 [85]
Guar gum	Bread + beverage	30 healthy	5 g	Reduced peak (45 min)<br>Reduced AUC	Not evaluated	Wolf et al, 2003 [16]
Guar gum + whole-grain corn flour with high amylose content	Maltodextrin	20 healthy	~ 15 g (dietary fiber)	Reduced AUC postprandial (240 min) and second meal in 2 products (cereal bar and smoothie drink)	Reduced AUC postprandial (240 min) and second meal in 2 products	O'Connor and Campbell, 2016 [80]
Guar gum + Alginate	Corn flakes bars	48 healthy	5.5 g<br>1.6 g	Reduced AUC = 33%<br>Reduced peak = 30%	Not measured	Williams et al., 2004 [76]
Guar gum + whey protein (preload)	Glucose	18 healthy olders	4,4 g + 16,4 g 27	Reduced plasma glucose<br>Difference significant: 30, 45, 60 min<br>Reduced peak (mmol/L in 60 min): 3.7 ± 0.4 vs 4.3 ± 0.3	Increased plasma insulin (30 min):<br>18.1 ± 2.4 vs 10.9 ± 1.5<br>colocar no texto	Pham et al., 2019 [83]
Partially hydrolyzed guar gum (HG)	Control = normal diet<br>Time zero	45 T2DM (n=22 control,)	10 g (HG) for 4 and 6 weeks	HG group vs control<br>Fasting plasma glucose (mmol/l) 7.4 vs 7.9<br>HbA1c (%) 6.8 vs 6.9	Not evaluated	Dall'Alba et al. , 2013

Table 2. Characteristics of clinical studies with significant responses on glycemic and insulin response

Guar gum (G)<br>Whey protein (W)<br>Guar gum + whey protein (WG)<br>(preload)	Mashed potato meal (C)	21 with T2DM	5 g<br>17g<br>5 g + 17 g	Reduced blood glucose<br>W and WG vs C (30, 60, 90, 180 min)<br>G vs C (30, 180 min)<br>WG vs G (60, 90 min)<br>Reduced aUC WG vs G	Reduced W vs C (30, 90 min)	Watson et al., 2019 [82]
Partially hydrolyzed guar gum (HG) + highly cooked potato (HP)<br>HG<br>HP<br>(preload 30 min (P))	Rice meal (R)<br><br><br><br>R+HP<br>R+HG	17 healthy females	50 g available carbohydrates<br>(15 g from potato in R+ HP)<br>~12 g HG in 100 g water	Reduced blood glucose<br>PHG+PHP+R or PHP+HG+R vs R and PHP+R (30 min);<br>PHG+PHP+R vs PHP+HG+R (30 min)<br>Reduced peak in 15, 30, 45 min,<br>and delayed peak, higher glycemic increments at 60, 90, 120 min<br>PHG+HP+R vs R, HP+R, HG+HP+R	Not evaluated<br>Not measured?<br>padronizar	Zhao et al., 2020 [84]
Galactomannan + inulin + β-glucan (1:1:1)	2.0 g/day (Insoluble dietary fiber)	30 with mild hyperglycemia and visceral fat accumulation	7.5 g/day DF (12 weeks)	Reduced fasting glucose = -0.5 mmol/L (6.47 vs 5.96)	Reduced Fasting insulin = ~20 nmol/L<br>Reduced HOMA 3.2 to 2.2	Kobayakawa et al., 2013 [77]
Konjac galactomannan (KGM)	OGTT (75 g glucose)	20 T2DM	GM (1 g) - preload<br>GM (3 g) for 4 weeks – Long-term	Preload - Reduced rise blood glucose in OGTT<br>Long-term - Reduced glucose AUC in 120 min	Preload - No reduced rise blood insulin<br>Long-term - Reduced HOMA-IR	Chearskul et al., 2007 [88]
KGM	Placebo noodles (PN)	32 with metabolic syndrome	KGM Noodles (400 g – 1 GM) for 4 weeks	GI (n=10) = KGM Noodles (34) vs PN (77)<br>No reduced in fasting blood glucose<br>Reduced HbA1c in T2DM (n=24)	No reduced in fasting blood insulin and HOMA-IR	Cheang et al., 2017 [89]
KGM+Xanthan gum<br>Guar gum+Xanthan gum	Beverage no added	20 healthy men	KGM+Xanthan gum (90:10) – 0,29%<br>Guar gum+Xanthan gum (50:50) – 0,44%	No reduced glycemic response	No reduced insulin response	Paquet et al., 2014 [79]
Glucomannan flour (GMF)	Soy milk ice cream (control)	9 healthy	Soy milk ice cream with 2.5 % GMF (SIC2.5%)	Reduced glycemic response<br>SIC2.5% vs control<br>GI = 56, GL = 9 vs GI = 76, GL = 12	Not evaluated	Sa'adah et al., 2018 [106]

Table 2. Characteristics of clinical studies with significant responses on glycemic and insulin response

Pectin (PTD)<br>Cellulose (CED)	Fiber-free control diet (CD)	8 healthy women	10 g PTD<br>10 g CED	PTD vs CD and CED vs CD – Reduced AUC<br>PTD vs CED – no difference	Not evaluated	Lee et al. 2006 [91]
Pectin from fruits of the date palm (different cultivars)	Glucose	at least 6 healthy	50 g carbohydrates	Itima GI = 31<br>Aliwrached GI=36<br>Tinissine GI = 37<br>Bentqbala GI = 42<br>Ghars GI = 49<br>Mizit GI = 52	Not evaluated	Yamina et al., 2021 [92]
Pullulan	MD	28 non diabetic adult	50 g added at beverages	Pullulan vs MD - Reduced incremental peak (mmol/L) 4.24 ± 0.35 vs. 1.97 ± 0.10 and iAUC (50%)	Not evaluated	Wolf et al., 2003 [16]
Pullulan	MD	34 healthy	50 g added at beverages	No significant effect	Reduced incremental peak insulin (23%) and AUC (20%)	Spears et al., 2005 [93]
Pululan<br>Pululan + SCF70<br>SCF 70<br>RS 75<br>RS 60<br>RS +SCF 70<br>Soluble Fiber Dextrin (SFD)	2 control meals (50 g of glucose)	12 healthy	25 g DF	Fibers vs control – Reduced iAUC at all times<br>iAUC SFD (mmol × min/L) = 50.1 ± 6.9 (value much higher than the others) vs 8.7 ± 4.1 to 28.5 ± 5.6	iAUC SFD (mUI × min) = 449 ± 120 (value much higher than the others) vs 50 ± 29 to 161 ± 52	Kendall et al., 2008 [28]
Pullulan<br>Long-chain (LCP)<br>Medium-chain (MCP)	MD	12 healthy/group	15 g	Reduced excursion glucose: LDP<MSP<MD	Reduced excursion insulin: LDP<MSP<MD<br>Reduced AUX (mmol × min) = LCP (11.32), MCP (15.73) and MDX (17.90)	Peters et al., 2011 [94]
RMD<br>(tapioca - TRM)	Glucose (GL)<br>Tapioca maltodextrin (TM)	16 healthy	TRM<br>MIX15 (7.5 g TRM + 42.5 TM)	Reduced plasma glucose (mg/dl)<br>TRM (104.60 ± 2.63) vs GL (135.87 ± 4.88), TM (127.93 ± 4.05), MIX15 (124.67 ± 5.73) MIX50 (129.33 ± 5.23)	Reduced plasma insulin (µIU/ml)<br>(13.01 ± 2.12) vs GL (47.90 ± 11.93), TM	Astina; Sapwarobol, 2020 [101]

Table 2. Characteristics of clinical studies with significant responses on glycemic and insulin response

			MIX50 (25 g TRM + 25 TM)		(52.96 ± 17.68), MIX50% (33.16 ± 4.99)	
RMD (tapioca - TRM)	Oral nutrition supplements (ONS)	17 healthy (acute evaluation)<br><br>22 healthy (12 weeks)	TRM15 (15 % TRM replacement)<br>TRM30 (30 % TRM replacement)	Reduced HbA1c% after 12 weeks TRM30 (5.2 ± 0.07) vs (baseline 5.5 ± 0.07)	AUC insulin TRM30 (33.12%) vs TRM15 (12,97%) and ONS – acute study	Astina et al., 2022 [100]
Nutriose	MD	120 overweight men	34 g/day (12 week)	Reduced HbA1c (- 0.45 ± 0.15%) in individuals with metabolic syndrome	Reduced HOMA = 18%	Li et al., 2010 [19]
Nutriose	MD	65 females T2DM	10 g/day<br>8 weeks	Reduced fasting plasma glucose (9.8%) Nutriose vs MD	Not evaluated	Farhangi et al., 2019 [97]
Nutriose	MD	36 normal weight+overweight	14 g/day<br>Preload/28 days	Reduced glycemic response (mmol/L) in mid-morning in day 14 (88.3 ± 4.6) and day 28 (88.1 ± 4.6) vs baseline (95.3 ± 4.9) and (93.3 ± 4.7)	Not evaluated	Hobden et al., 2021 [98]
Soluble corn fiber (SCF)	Maltodextrin (MD)<br>Glucose	22 healthy	~25 g SCF (glutinous rice + SCF and beverage +SCF)	Reduced iAUC relative to MD and glucose<br>Rice - reduction at all studied times<br>Beverage - reduction at 55 and 100 min	Reduced iAUC in relation to MD and glucose<br>Rice - reduction up to 70 min<br>Beverage – reduction between 40 and 130 min	Tan et al., 2020 [102]
SCF<br>Polidextrose (PDX)	Diets: FULL energetic and ISO energetic	18 overweight	55 g SCF<br>57 g PDX	SCF or PDX vs FULL – Reduced peak after breakfast<br>SCF vs FULL = FULL – Reduced peak after second meal	SCF or PDX vs FULL – Reduced AUC after breakfast and second meal	Konings et al., 2014 [28]
Glucomannan + xanthan gum (7:3)	Biscuit without DF<br>Wheat bread,<br>Bread with 12 g margarine	10 healthy<br>9 T2DM	10 g (in biscuits)	Reduced GI<br>GI (healthy) = 26<br>GI (DM2) = 37	Not evaluated	Jenkins et al., 2008 [103]

Glycemic index (GI); Insulinemic index (II); Area under the curve (AUC); Incremental area under the curve (iAUC); Dietary fiber (DF); Prowashonupana Barley (PW); Barley flour (BF); Sieved fraction (SF); Water-extracted fraction (WF); High molecular weight (HMW); Low molecular weight (LMW); Glycated hemoglobin (HbA1c), homeostasis

Table 2. Characteristics of clinical studies with significant responses on glycemic and insulin response

model assessment (HOMA); Diabetes type 2 (DM2); Oral glucose tolerance test (OGTT); Glycemic response (GR), Insulinemic response (IR); Body mass index (BMI); Oat β-glucan (OBG); Barley balance (BB).
